# The Magical and the My-Person in Psychoanalysis During the Covid Pandemic

**DOI:** 10.1177/0003065120981733

**Published:** 2021-01-13

**Authors:** Paola M. Contreras

**Keywords:** Covid, dream thinking, the social, magical thinking, personal, online, transformational thinking, my-person

When the United States Center for Disease Control (CDC) declared the novel coronavirus’s spread a pandemic, I wondered what might come next. Before the CDC announcement, I was in an active state of denial. Sometimes I thought the constant chatter in the news about the virus an exaggeration. In the couple of weeks before the governor ordered my state of Massachusetts into shutdown, I went to a large dinner party with psychoanalyst friends. I attended seminars at my institute, taught classes, and continued to meet with all my patients.

I recall that on one of those days leading up to the shutdown, I was returning to my office from an impromptu lunch at a local restaurant, which would typically have been impossible to visit without a reservation. The streets were already emptying, and I felt relieved by the quieting of the everyday bustle. I saw a colleague in the lobby who looked dazed. She expressed fear about feeling unwell and worried that she might have the virus (we later learned she did not). She was mostly terrified that she might have infected others. She told me she would not attend our evening seminar and asked if I could bring her into the meeting via Zoom. Only she and I were to attend that class, as our other colleague was out of the country. Still in denial, I recall feeling slightly annoyed that I would be the only candidate attending that evening’s meeting in person.

As I put these thoughts in writing, I am sitting in a home office where since March 16 I have been meeting with patients over Zoom or by telephone. Today is July 23, and I don’t yet know when I will resume in-person sessions. Looking back, I was clearly in denial through those early days when coronavirus began its steady spread through Massachusetts. A Biogen conference in Boston had been one of the state’s super-spreader events. I remember that I read the news and felt irrationally distant from it sitting in my Cambridge office, only six miles from where the Biogen employees had met.

In what follows, I will discuss thoughts that have shaped my ideas about the Magical in psychoanalysis, thoughts arising from my experience as an analyst practicing during the Covid pandemic. I will start by introducing a concept, the analyst’s *my-person*, that frames why the analyst’s personal experience is relevant in the work of being an analyst, particularly in times of unrest. I will then review some of Ogden's ideas on thinking (2010) and relate them to what I call the Magical in psychoanalysis. I will distinguish the Magical from Ogden’s magical thinking and relate it to his thoughts on dream thinking and transformational thinking. To further explain the Magical in psychoanalysis, I will explore examples of magical realism in literature.

Finally, I will outline how I think about the Magical as a transformational process that emerges when the analyst finds ways to transform the “my-person” in order to continue working through great unrest.

## The Analyst’s My-Person

As a psychoanalyst who asks that her patients situate themselves within their own experience to speak with me, I need to do something similar when speaking to the public. That is, the psychoanalytic public, to cognitively and emotionally follow what I say, need to know something about who is speaking to them.

Covid and the social unrest that has unfolded amid the pandemic in response to George Floyd’s murder by police in Minneapolis is uncharted territory for many psychoanalysts in the United States. Theory intertwines with the theorist's personal experience. I am invoking here Bion’s invaluable contributions on learning from experience. Beta elements, the unprocessed, will require an alpha function (i.e., a parent, an analyst, a containing other) to metabolize the unprocessed thing into a dream, a memory, a thought, or anything that can help the mind continue to move forward. Alpha is then the analyst’s tool to help the analyst and the patient become “unstuck.” When I share how my mind deals with the current Covid crisis, I show the reader how alpha works for me as an analyst and how I use it in work with patients.

I share personal background simply because I have no choice but to bring myself to the context. I call this process of bringing myself into my thinking as a psychoanalyst the *my-person*. My-person has developed alongside my becoming and being an analyst. I use *my-person* rather than *myself* to highlight that I am speaking about something away from the conscious self and closer to the unconscious, which typically remains personal in the consulting room. In other words, psychoanalysts typically disclose less frequently compared to other mental health professionals.

My-person makes sense of what I encounter in everyday life (say a conversation with a friend about an issue) or the things I do (participate in a protest). My-person contributes, to some degree, to the continuous shaping of history. Thus, to be a psychoanalyst who is involved with and responsive to the sociopolitical, it is insufficient to limit my-person to quoting theory concerning the events I live and witness. My-person needs to find a way into my work as a psychoanalyst to make the work possible.

When I am a psychoanalyst in the clinic, I describe my patients and their story, relate it to the theory, and sometimes generate new concepts. I can—and it is desirable to a certain extent that I do—omit most of my-person when I write about my work with a patient. There, in the clinic, my patient is the subject. To approach a social issue, I must start with my-person because I am one of the many subjects of that story. All psychoanalysts pushed onto a computer screen with their patients are the subject of the being-a-psychoanalyst-amid-Covid story. Each of us has a story to tell.

Bion (1997) kept a diary during combat in World War I that he published and that became known in psychoanalysis as the theorist’s war memoir. The diary was about his years of being a tank commander in France. An impressive story is Bion’s account of Sweeting, a runner who accompanied Bion into combat and was ripped open by shrapnel that left his lung exposed. As Sweeting faded into his death, he begged Bion to remember his mother’s address and to write to her. Bion began to vomit and yelled at the soldier several times to shut up. Bion wished the soldier would die, probably to alleviate the horror he was feeling.

Various authors have made connections between Bion’s trauma and his psychoanalytic theory (Mawson 2011; Altman 2016; Souter 2009). For instance, Altman suggests that Bion subliminally encoded the trauma of war in his theory and practice, and Souter outlines how Bion, a man traumatized by war, used the experience to create a theory of trauma. The need to know is central in Bion’s theory, and to know we need to think, and to think we need to put things together—Bion’s well-known links. Trauma interrupts and might even destroy the ability to link.

Symington and Symington (1996) conjecture that Bion’s war writings hold the embryonic analyst in him: “We may conjecture that it was through a knowledge of his soul that he came to understand so well the psychotic in the personality. It was also the seed of his later mysticism” (p. 22). Interestingly, Bion never felt compelled to conjecture on the connection between his psychoanalytic concepts and his personal experiences the way theorists who have studied his work were to do. I venture to say that Bion’s in-depth unconscious knowledge about the irreducible nature of the *thing* that makes the analyst an analyst may have kept him from making such conjectures himself. However, he did lay out all his parts to us: some of the most extreme personal experiences in his diaries, and volumes of writing where he describes his theories. And to my knowledge, he never asked that people not relate his concepts to his personal experiences. Bion challenged analysts to practice radical transparency about their thinking and feeling. “If it is possible to isolate a central message which Bion bequeathed to posterity,” remark Symington and Symington (1996), “it would be: *Think and speak from your own heart and mind.* He addressed his mind to an analysis of experience. Naturally this meant his own personal experience” (p. 14).

Hence when I speak of the my-person, I am less interested in cognitive conjectures linking the analyst’s personal experiences and the analyst’s theorizing. From the my-person, I am most interested in exploring the following question: What do any psychoanalyst’s personal experiences evoke? More specifically, because the my-person of the analyst will also be present when at work with the patient’s unconscious, what do the analyst’s personal experiences evoke amid the process of being a psychoanalyst? And what do the my-person experiences I will share here with the reader evoke? What have they to do with the experience of being an analyst in the time of Covid?

Readers will have to draw their own conclusions.

## My-Person Thoughts

A my-person memory that mirrors how I moved through the initial days leading to the Covid shutdown is from the time I moved back to Guatemala with my family at the age of ten. My parents uprooted us from the Chicago area, where I was born, and where they had found refuge from a war-ridden Guatemala. When we arrived in Guatemala, there was still active conflict and government repression, and it would take several more years to sign the 1996 peace accords that ended the country’s thirty-six years of civil war.

Chaos plagued Guatemala in a manner that stood in sharp contrast to the quiet of the northern suburbs of Chicago, where I spent much of my childhood. Soon after our arrival, I recall experiencing an initial shock witnessing violence in Guatemala. I spoke about this event with my analyst in Cambridge nearly thirty years later, experiencing, probably for the first time, all the feelings tethered to the memory. It was of me as an eleven-year-old girl going with my father to a local cockfight. I have always loved animals. I was excited, thinking I would see roosters. Instead, I was stunned by a horrifying scene I witnessed from the creaky wooden bleachers where I sat.

Two roosters, one with reddish feathers and the other with yellow tones, propelled by their instinct to fight, ran out to the center of the cockpit ring. Unbeknownst to the roosters (and me), their owners had tied metal blades to their spurs, causing each kick to inflict a gashing wound. Blood flew through the air several times, and within seconds the referee declared one bird the winner when the other’s beak plunged to the ground as it died.

I wept some silent tears and swallowed hard not to sob—the people there, mostly men, where enthralled with the spectacle. I intuitively knew that their bravado, including my father’s, would overwhelm my grief if I let it show. The violence of the cockfights foreshadowed the violence I frequently witnessed over my years in Guatemala. Seeing dead bodies became almost normalized. Some of the bodies were of people who had been hit by cars on high-speed roads that doubled as pedestrian ways for small villages. More than once I saw the dead bodies of people who had been tortured, left out on the road close to my grandmother's home in a town on the country’s south coast near the Mexican border.

Secret government operatives left these tortured bodies out as warnings, I learned, to instill fear and show the grave punishment meted out for subversive left-leaning activity. For the first time, I understood what it meant when my mother told me that her father and sister had been tortured and executed by the Guatemalan military. My grandfather, a congressman, had been accused of communist activity when he pursued water rights for a local Mayan community. His daughter, only sixteen at the time of their murder, was an unfortunate casualty who had accompanied him that day.

## Different Forms of Thinking and the Magical

Ogden (2010) has noted that psychoanalysis can focus on *how* a person thinks or on *what* a person thinks. Different ways of thinking include magical thinking, dream thinking, and transformative thinking.

Magical thinking relies on omnipotent fantasy to create an internal reality that feels more real and tolerable than external reality. Magical thinking is a defense, a place of rest from an overwhelming external re-ality. However, it does not work over the long term because nothing, except other magical constructs, can be built on it.

Dream thinking is related to the process of dreaming that happens both in waking life and during sleep. According to Ogden, dream thinking is our most profound form of thinking, is mostly unconscious, and interacts with preconscious and conscious thinking. Dream thinking promotes psychological growth, and at some point needs to be carried out with another to dream think together. Transformative thinking is a form of dream thinking that leads to a radical change in how a person organizes experience. Transformative thinking recognizes the limitations of categories of meaning that have been thought of as the only ones and creates new ones “unimaginable up to that point” (p. 334).

I think that in the early days of Covid I resorted to various forms of magical thinking. I could not see the reality—that there was a virus in a steady spread that would eventually affect everyone. I think that having been exposed in Guatemala to an overwhelming fear I could not physically escape, I learned not to see what I saw, the violence. In other words, I learned to see the violence and not take it all in. Alternatively, I took it in but quickly tucked it away, far out of consciousness. Once I brought these experiences to my analysis, I transformed them and used what I learned about them in my own work as an analyst. Perhaps my initial reaction to the pandemic helped me stay calm, move on through my day, and deal with all the affairs that would need tending to with my family and work in the days that followed. I efficiently closed my office in the eight-story building where I saw my patients and set up shop at home. However, to carry myself and my patients through the transition and find a way back to our analytic work, I also needed to move out of that magical thinking place where everything was seemingly okay.

Over time, the Covid environment brought me to wonder where and what *the* Magical is in psychoanalysis. I want to emphasize that I am not talking about magical thinking. Now I am referring to *the* Magical, which is some of the substance that may lead the analyst to Ogden’s two other forms of thinking—dream thinking and transformative thinking—during times of unrest.

Covid has changed the way psychoanalysts practice. Before the pandemic, I was in my physical office, and during sessions I sat in my chair and my patients would sit or lie on a couch looking away from me. Now I sit in a chair in front of a screen in my home, or wherever I am. My patients sit wherever they want to in their home, or wherever *they* are. A significant part of the frame, my office, and the oft-fetishized couch is now a mere box on a screen that projects my image and lights up when I speak, and another box that projects my patients’ image and lights up when they speak.

To bridge further into the Magical of psychoanalysis in the time of Covid, I want to talk about magical realism in literature. This is a genre in which the author describes the mundane with detailed and elaborate descriptions while revealing the magical in the existing world (Zlotchew 2007). Magical realism is not fantasy, because the writer does not invent a new world. Instead the magical realist author describes the existing world, but many of the occurrences defy explanation. The Mexican critic Luis Leal (1995) notes that if a narrated occurrence can be explained, it is not magical realism. “To me,” he writes, “magical realism is an attitude on the part of the characters in the novel toward the world” (p. 128). Magical realism became a worldwide phenomenon popularized out of Latin America (Parkinson Zamora, and Faris 1995) from authors from countries facing great unrest—Argentina, Colombia, Cuba, Mexico, and Venezuela (a list not meant to be exhaustive). Themes related to political unrest and timely social issues are a common feature of magical realist literature. It is reasonable to assume that an essential aspect of magical realism is the author’s capacity to transform, as Ogden would suggest, the thought-to-be-unmetabolizable experience into a palatable and captivating narrative.

Just as magical realism both is and is not a story about what really happened, so in analytic work patient and analyst transform a session’s content, which over time becomes something that is and is not what really happened. That is, the patient generates a new self-narrative through the work of analysis, which continues to hold the essence of the original story. In this working through and transformation of the patient’s narrative, the analyst needs to be involved in varying degrees, depending on the theoretical perspective that informs the work. However, I believe that in times of great social unrest part of this transformation requires that the analyst work from the personal, the my-person. The analyst will need to become more involved and push the analytic frame, adapting it to meet the new circumstances.

Hence, the Magical of the session will come through various forms of dream thinking and transformative thinking via the analyst’s my-person involvement in the work. I will say more about the Magical of the session later. But first I want to share an example of magical realism in literature pertinent to Covid, which I will use to bridge a patient’s dream.

## The Magical in Literature and in the Session

Gabriel García Márquez’s *One Hundred Years of Solitude* is one of the quintessential books of magical realism. It is the story of seven generations of the Buendía Family in Macondo, a utopian village. A recurring theme is the town’s inescapable repetition of history. All the characters are destined by their past and by the complex movement of time. In the novel, time can become confusing; the past can feel like the present and vice versa. The future is always uncertain.

During Covid, I have at times felt like a character in a magical realist novel. Every weekday, I turn on Zoom and sit in a makeshift office pulled together in my home following the shutdown. I surround myself with Zen-like panels and a hardy snake plant, the only thing brought from my former office into the new one. In womb-like fashion, Covid sequesters me behind a cacophony of elaborate sound machines meant to drown out the sounds of my lively home.

García Márquez describes a plague in *One Hundred Years of Solitude*, a plague of insomnia. It is brought to Macondo by Rebeca, a little girl who one day appears out of nowhere. The insomnia plague starts with an inability to sleep, followed by memory loss that eventually reduces to idiocy all who contract it. Soon enough, several of the Buendia family are infected and cannot sleep for days. They dream while awake. The Buendias, in turn, infect the entire town.

The insomnia plague that García Márquez describes is an actual condition, fatal familial insomnia (FFI) described by Sghirlanzoni and Carella (2000). A rare genetic degenerative brain disorder, it is characterized by insomnia with a mild onset that progressively worsens, ending with significant mental deterioration.

The magical realist’s art is to translate the most terrifying of experiences, like a plague or pandemic, into the ordinary. The genre should not strike analysts as strange; this, after all, is what the unconscious does. It makes the extraordinary (e.g., the Covid experience) into, say, an ordinary dream that the patient talks about in a Zoom meeting. I, as psychoanalyst, then help translate it.

“I had a dream,” my patient began. I had been treating him, a man in his late thirties, for six months. The session I am describing was one of the first three I conducted over Zoom following the shutdown, and I was still quite uncomfortable using the technology. I remember that I was working hard to look at his face and the green light on my screen so that he could see (or feel like) I was looking at him. Later I would learn to hide my image from the Zoom screen, to avoid the disorientation of seeing myself (not my-person) on the screen. Was it my patient there, in the image of him that I saw? Was I here, where I was sitting, or there, on his screen? Where did we exist?

Thoughts like this visited my mind in the early days of my experience of being-a-psychoanalyst-amid-Covid. I still have these questions, but they have faded some as I have become more comfortable. At the beginning of the shutdown, I recall having more personal conversations with my patients at the start of sessions. There was a stabilizing that was happening for them and me. One way to understand this abrupt license of self-disclosure was that my-person needed to be more present because this was a new experience that affected my patients and me. In order to get to a place where we could both dream think again, their personal experience with Covid and my my-person experience with it needed metabolizing.

Now back to my patient’s dream. Someone had taken an embarrassing video of him doing something. “I can’t recall,” he said, “what I was doing in the video, but everyone was talking about it. I was mortified and finally mustered the courage to ask a friend to show me the video. When I saw the image, I started yelling in horror, ‘Where are my feet? I couldn’t see my feet!’”

One way to talk about this dream is to follow its symbolic content, as Ogden explains. He proposes that it is also possible to follow the process of how the dream is thought. The symbolic way to deal with the dream is, for example, to explore the missing feet. Does it have something to do with the phallic, and is it about castration? Is it something obsessive, perhaps? The other way is to live the dream with the patient. However, this path is not accessible via our thoughts. As phenomenology highlights (Merleau-Ponty 1945), our thoughts are at the tail end of an elaborate process called experience, which is the becoming, the burgeoning, the flowering of what is to come next. Bion showed us this. I purposely say that he “showed” us this, rather than that he taught or explained it, because reading Bion often feels like an experience rather than a cognitive exercise. The other way, then, the noncognitive, noninterpretive way, is to live the dream with the patient—dream think.

Where are *my* feet? I thought after my patient talked about this dream. Where are your feet? I am asking you, the reader. Covid has been a time marked by uncertainty. None of us living through this experience have our full footing. Throughout this experience, and with the more recent events of George Floyd’s murder and the ensuing protests and riots, we have all, at times, lost our sense of internal and external footing. Further, on these Zoom screens, we have literally lost our feet, we have lost our legs, we have lost our genitals. And I am talking about us, we psychoanalysts, where typically we would be talking only about them, our patients.

During the pandemic, one option has been for analyst and patient to seclude themselves in fantasy and ignore the outside, to deny reality. Some dyads might believe that the virus is not real, that it is a hoax, and that we are immune. In the session, we could also flee and seclude ourselves in the deep unconscious in an attempt to deny the reality that, every day, people are dying from the virus. I know I went into flight in the days leading up to the shutdown, a reaction likely evoked by the my-person experiences I described earlier. It was only after the shutdowns that the reality of Covid became explicit in my sessions with patients. Another relevant my-person experience was that sometime after witnessing the cockfight, I recall, I opened the cages of an uncle’s fighting cocks. I let all the birds loose and then hid in a nearby lime tree orchard. From afar, I could see someone running after the freed roosters while I desperately hoped he would not recapture them. Was this act of freeing the birds the burgeoning psychoanalyst in me? Before I became an analyst, training as a psychologist in Guatemala, I was inspired by the work of Ignacio Martín-Baró (1996), whose liberation psychology theory has gradually become known by analysts in the United States. I carried these social justice roots with me into my becoming a psychoanalyst.

With exclusive focus on the unconscious, we can try to deny that amid the Covid shutdowns people keep losing their jobs and homes and that the most vulnerable are going hungry. We can try to forget that undocumented people, so heavily relied on to care for our children, clean our homes, keep our lawns, and harvest and process our food, have been disproportionately affected. Or to forget that the U.S. Department of Homeland Security continues to separate families at the border. And that our government unleashed secret police forces to terrify U.S. citizens peacefully protesting racial injustice—an action that inevitably reminds me of Guatemala’s repressive government through the 1990s. How do I authentically settle back into practice bringing this new reality with me? How do I start to feel enough at ease with my new reality to look for ways to refashion the frame of my analytic work? At the same time, how do I not relegate the work of analysis to a form of denial and magical thinking? Through these times of unrest, how do I promote, instead, dream thinking and transformative thinking that leads to the Magical potential of the session?

The Covid pandemic has been our field’s equalizing event. It has also been a decentering event that has made it possible for us to witness one of the places where the Magical of psychoanalysis can appear. Ogden, discussing transformative thinking, merits direct quotation:

We, as psychoanalysts, ask of ourselves and of our patients no less than transformative thinking even as we recognize how difficult it is to achieve. Our theoretical and clinical work becomes stagnant if at no point do we engage in transformative thinking. It is this striving for transformative thinking that makes psychoanalysis a subversive activity, an activity inherently undermining of the gestalt (the silent, self-defining terms) of the intrapsychic, the interpersonal, and the social cultures in which patient and analyst live [2010, p. 335].

The South American field theory that has interested many U.S. psychoanalysts lately (Lisman-Pieczanski and Pieczanski 2015) was created by people who had escaped nations at war or lived in places marked by great unrest. Madeleine and Willy Baranger left France amid World War II and moved to Argentina. I assume that analysts like the Barangers, living through challenging times, had felt they had lost their footing. Mimi Langer fled Vienna for Argentina during the war, only later to seek political asylum in Mexico to escape Argentina’s Dirty War. She generously tells us, through Nancy Caro Hollander (1997), how she lost her footing not once, but several times, and how those experiences deeply informed who she was as a psychoanalyst.

Collective unrest, the extraordinary erupting through the ordinary, provides an opportunity for creativity in many places and spaces, including psychoanalysis. It is a process that mirrors the work of the unconscious, the unconscious being the extraordinary that erupts into ordinary conscious life. When we experience the extraordinary as a group, as analysts across the globe are experiencing it right now, it shifts reality into an unreality. We wonder if we are awake or asleep, or are in a dream. In other words, we begin to, in a sense, walk and live in the unconscious. Suddenly, there appears the opportunity for reality and fantasy to be the same.

In García Márquez’s novel, the figure of Melquíades is central. He is a gypsy who visits Macondo to share objects of science and of magic. The town is eventually saved by him when he gives everyone a potion, a vaccine of sorts, that revives all memories. He dies and revives many times in the book, each time returning to guide a new generation of the Buendia family. I think of psychoanalysis as the figure of Melquíades. It must die or almost die, then revive, connect, and deal with reality, transform and offer a new proposal that bears the old, welcomes the new, and eagerly plunges forth into the future. This process is the Magical of psychoanalysis and the Magical that the analyst can offer. It shows up most clearly amid unrest, whether social or intrapsychic.

In the social, the Magical in psychoanalysis is Gillian Straker working during apartheid in South Africa and attending to people being politically persecuted (Fidler and Kanowski 2019). The Magical is Mimi Langer working with refugees in Mexico while she herself was one. The Magical is Beverly Stoute delivering during APsaA’s first virtual conference a riveting plenary that formulated Black rage following the videotaped police murder of George Floyd that traumatized the globe and inspired and provoked protests and riots worldwide.

The Magical of psychoanalysis reveals itself when the analyst, amid great unrest, can keep working, can stay present, remain and be a person, and bridge back to functioning as an analyst to work again toward transformative thinking with the patient. It is in the struggle to get back to work that the Magical of psychoanalysis lives.

Who are we becoming as analysts as we live through these Covid times and through these times of social unrest? The bigger question is, will psychoanalytic leaders let the new that is emerging come through? Will they invite the change it will demand? Will psychoanalysts be so bold as to let what the Magical offers permeate our practice?

What if this is *the* opportunity for the Magical to emerge in U.S. psychoanalysis? I hope we will heed the call and transform ourselves, à la Ogden.
